# Realizing High Thermoelectric Performance at Ambient Temperature by Ternary Alloying in Polycrystalline Si_1-x-y_Ge_x_Sn_y_ Thin Films with Boron Ion Implantation

**DOI:** 10.1038/s41598-019-50754-4

**Published:** 2019-10-04

**Authors:** Ying Peng, Lei Miao, Jie Gao, Chengyan Liu, Masashi Kurosawa, Osamu Nakatsuka, Shigeaki Zaima

**Affiliations:** 1 Department of Materials Physics, Graduate School of Engineering, Nagoya University, Nagoya, 464-8603 Japan; 2 Guangxi Key Laboratory of Information Material, School of Material Science and Engineering, Guilin University of Electronic Technology, Guilin, 541004 China; 3 PRESTO, Japan Science and Technology Agency, 4-1-8, Honcho, Kawaguchi, Saitama 332-0012 Japan; 4 Institute for Advanced Research, Nagoya University, Nagoya, 464-8601 Japan; 5 Institute of Materials and Systems for Sustainability, Nagoya University, Nagoya, 464-8601 Japan; 6 Institutes of Innovation for Future Society, Nagoya University, Nagoya, 464-8601 Japan

**Keywords:** Thermoelectric devices and materials, Thermoelectric devices and materials, Thermoelectrics, Thermoelectrics

## Abstract

The interest in thermoelectrics (TE) for an electrical output power by converting any kind of heat has flourished in recent years, but questions about the efficiency at the ambient temperature and safety remain unanswered. With the possibility of integration in the technology of semiconductors based on silicon, highly harvested power density, abundant on earth, nontoxicity, and cost-efficiency, Si_1-x-y_Ge_x_Sn_y_ ternary alloy film has been investigated to highlight its efficiency through ion implantation and high-temperature rapid thermal annealing (RTA) process. Significant improvement of the ambient-temperature TE performance has been achieved in a boron-implanted Si_0.864_Ge_0.108_Sn_0.028_ thin film after a short time RTA process at 1100 °C for 15 seconds, the power factor achieves to 11.3 μWcm^−1^ K^−2^ at room temperature. The introduction of Sn into Si_1-x_Ge_x_ dose not only significantly improve the conductivity of Si_1-x_Ge_x_ thermoelectric materials but also achieves a relatively high Seebeck coefficient at room temperature. This work manifests emerging opportunities for modulation Si integration thermoelectrics as wearable devices charger by body temperature.

## Introduction

Taking advantage of the Seebeck effect, the thermoelectric generator (TEG) as a type of energy harvester can deliver an electrical output power by converting a heat stream flowing there through. Shrinking and integrating TEGs present exceptional advantages like simplicity, size and power scalability, adaptability to different temperature ranges and a long-time stability facilitated by the absence of mobile parts^1^. The wearable TEGs is, a logical fit to harvest power from the thermal energy of body and use the harvested power to operate portable electrical systems^2^. e.g. mobile and wearable electronic devices, reject the batteries replacement process forever.

Recently, ambient thermoelectrics have been developed very quickly, such as poly(3,4-ethylenedioxythiophene)-poly(styrenesulfonate) (PEDOT:PSS)^3–5^ and its composites with tellurium nanowires^6,7^ carbon nanotubes (CNTs)^8^, or metal-containing conducting polymer, poly[K_*x*_(Ni-ett)], which delivered an equally high power factor (PF) of up to 4.53 μW cm^−1^ K^−2^ and an encouraging *zT* value of 0.3^9^. etc. However, ambient thermoelectricity still struggles with the lack of low-cost, abundant, and environmentally friendly materials. With the possibility of integration in the technology of semiconductors based on silicon (Si) electronics, high harvested power density, abundant on earth, nontoxicity, and cost-efficiency, silicon germanium (Si_1-x_Ge_x_) alloy has attracted significant attention among other thermoelectric materials^10,11^. However, despite its high power factor, Si is inefficient at room temperature. The efficiency of TEGs for wearable using depends on the properties of thermoelectric materials at room temperature. The biggest challenge for Si based wearable TEGs is to achieve a high thermoelectric performance at an ambient temperature instead of its high temperature TE applications.

Three material properties, Seebeck coefficient *S*, conductivity *σ*, and thermal conductivity *κ*, are vital to the thermoelectric generation^12^, and *zT* = *S*^2^*σT/κ* is employed to evaluate the thermoelectric performance of materials,where *T* is the absolute temperature. The power factor (*PF* = *S*^2^*σ*) could be largely enhanced through the optimization of carrier concentration, convergence of electronic bands^13^, energy filtering effect^14^, and quantum confinement^15^, etc.

Nonetheless, polycrystalline Si_1-x-y_Ge_x_Sn_y_ ternary alloy is chosen over other materials, such as poly-Si, poly-Si_1-x_Ge_x_, and Bi_2_Te_3_ compounds. We previously reported the improvement of the thermoelectric performance of Ge_1-x_Sn_x_ with Sn incorporating into Ge^16^. In this study, we expect that Sn incorporation into Si_1-x_Ge_x_ and boron (B) ion implantation have been taken as innovated ways to improve ambient Seebeck coefficient and electrical conductivity simultaneously. A theoretical prediction for the thermal conductivity of Si_1-x-y_Ge_x_Sn_y_ ternary alloy has been recently reported^17^, however there are few experimental study of Si_1-x-y_Ge_x_Sn_y_ thin films for investigating thermoelectric properties. Moreover, the recent interest in Sn by its possibility to reach direct band gaps^18,19^, which is possible to improve the electrical conductivity materials and reduce the thermal conductivity of Si_1−x_Ge_x_ thin films.

Hence, in this study, polycrystalline Si_1-x-y_Ge_x_Sn_y_ ternary alloy film on insulator has been investigated to highlight its efficiency through ion implantation and high-temperature rapid thermal annealing (RTA) process. Significant improvement of the TE performance has been realized in B-implanted Si_0.864_Ge_0.108_Sn_0.028_ thin films after a short time RTA at 1100 °C for 15 seconds, the power factor is 11.3 μWcm^−1^ K^−2^ at room temperature. The introduction of Sn into Si_1-x_Ge_x_ not only significantly improves the conductivity of Si_1-x_Ge_x_ thermoelectric materials at room temperature but also provides a relatively high Seebeck coefficient possible by the increasing of grain size due to the liquid Sn in high temperature accelerating the growth of Si_1-x_Ge_x_ grain^20^. The method of ion-implantation and grain size increasing achieved by Sn doping into polycrystalline Si_1-x_Ge_x_ thin films, and our design not only boosts the thermoelectric application of Si_1-x_Ge_x_-based materials but also enables a synergetic strategy for designing thermoelectric materials with high thermoelectric performance.

## Results and Discussion

**Figure 1:**
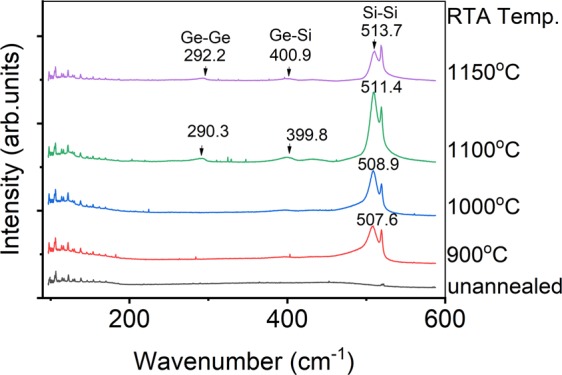
Raman spectra of the Si_1-x-y_Ge_x_Sn_y_ samples after 15 seconds-RTA.

Figure 1 shows the Raman spectra of the samples annealed at different temperatures for 15 seconds. The Raman spectrum of the ternary Si_1-x-y_Ge_x_Sn_y_ films looks similar to the Raman spectrum of binary Si_1-x_Ge_x_ with comparable Si contents^21,22^, and the four dominant peaks assigned to Ge-Ge, Si-Ge, and Si-Si modes from Si_1-x-y_Ge_x_Sn_y_ alloys and the Si substrate which are approximately observed at 290, 400, 510, and 520 cm^−1^ respectively. However, four visible peaks of ternary Si_1-x-y_Ge_x_Sn_y_ alloy is observed in the sample only over 1100 °C-annealing as shown in Fig. 1. One can see a clear trend that the peak intensity increases with the RTA temperature, but the 1100 °C-RTA sample is an inflection point, the peak intensity becomes weak instead in the 1150 °C-RTA sample which indicates that polycrystalline Si_1-x-y_Ge_x_Sn_y_ thin film be synthesized well only at an appropriately high RTA condition at 1100 °C for 15 seconds in our samples. The peak shift of Si-Si and Ge-Ge peaks to a higher wavenumber indicate the Sn content in ternary compounds decreases with the increasing RTA temperature^23^. The results of laser Raman measurements indicated that suitable annealing parameter not only promotes the polycrystallization of Si_1-x-y_Ge_x_Sn_y_ ternary alloy but also affects the Sn precipitation.

**Figure 2:**
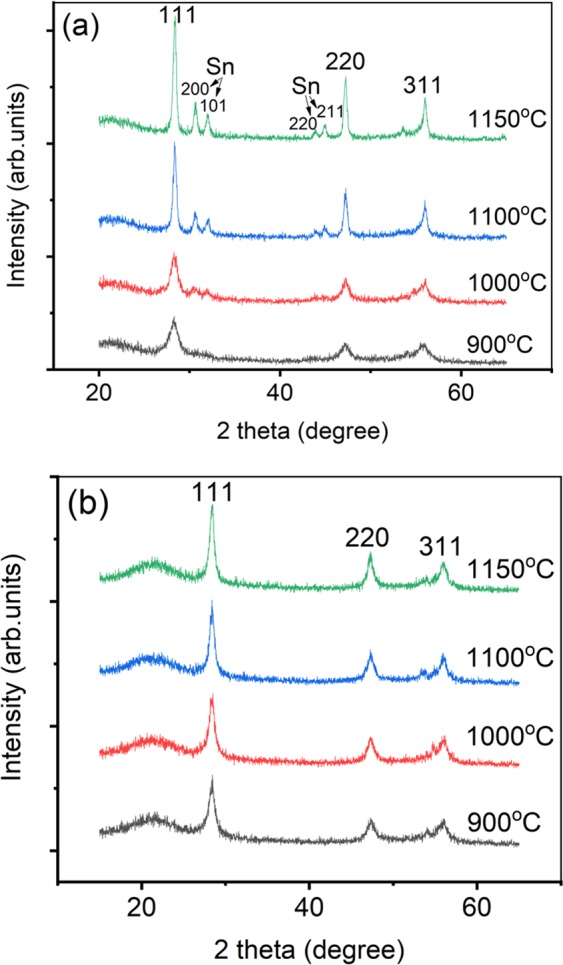
XRD profiles of (**a**) Si_1-x-y_Ge_x_Sn_y_ and (**b**) Si_0.889_Ge_0.111_ samples after 15 seconds-RTA.

In order to reveal the effect of the introduction of Sn on the ternary compound’s crystallization, we also prepared Si_0.889_Ge_0.111_ binary alloy samples with the same B dose for comparison. Figure 2(a,b) shows the XRD profiles of Si_1-x-y_Ge_x_Sn_y_ and Si_0.889_Ge_0.111_ samples, respectively, annealed for 15 seconds at different temperatures. Figure 2(b) show that the three higher intensity peaks locate at 2θ approximate 28.3, 47.2 and 56.0 degree which are similarly attributed to Si_1−x_Ge_x_ Bragg reflections of 111, 220, and 311, respectively^24^. Whereas, in Fig. 2(a) with the increase of annealing temperature, the peak intensity of Si_1-x-y_Ge_x_Sn_y_ increased more distinctly compared to Si_0.889_Ge_0.111_ samples, especially when the annealing temperature exceeds 1000 °C. Meantime, the diffraction peak related to β-Sn phase appears, especially obvious in the 1150 °C-annealed sample which means that even a short time of 15 seconds and high temperature annealing can cause part of the Sn precipitation.

The cross-sectional grain size (*g*) of the film could be calculated from the full width at half maximum (FWHM) of the diffraction peaks of the 111 Bragg reflection using the Scherrer formula *g* = *Kλ/Bcosθ*^25^ and the lattice spacing *d*_111_ can be calculated by the Bragg’s law 2*dsinθ* = *λ*^26^. Here *λ* is the wavelength (0.154 nm) of Cu K_α1_, *θ* is the angle satisfying Bragg’s law, and *B* is the corrected FWHM in radian, *K* ≈ 0.9. Generally, the crystallinity of polycrystalline Si_1−x_Ge_x_ thin film has been improved with the increase of annealing temperature^27^.

**Figure 3:**
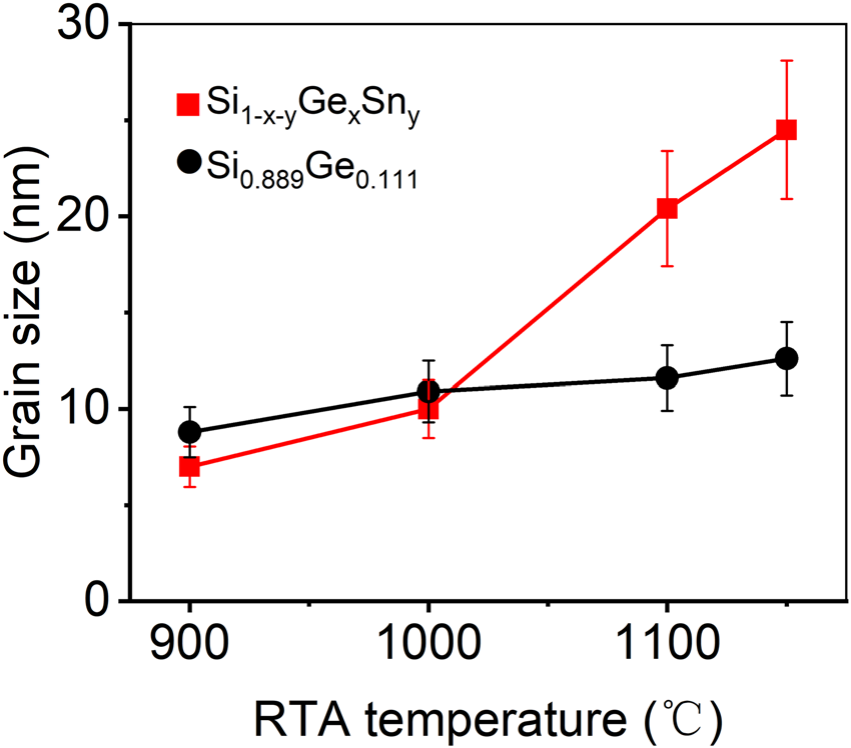
The RTA temperature dependence of the grain size for samples after 15 seconds-RTA.

Figure 3 shows the grain sizes in both Si_1-x-y_Ge_x_Sn_y_ and Si_0.889_Ge_0.111_ thin films increase with increasing annealing temperature, but the grain sizes increase in Si_1-x-y_Ge_x_Sn_y_ samples increases obviously faster than those of Si_0.889_Ge_0.111_ samples specifically when the RTA temperature over 1000 °C. Without doubt the 15 seconds shorter RTA process led to small crystallites diameters as well, as mentioned in the first section, the precipitated liquid Sn could prominently increase the grain size of Si_1-x-y_Ge_x_Sn_y_ in 1100–1150 °C range^20^, and perhaps the existence of high concentration boron atoms also attribute to rapid crystallite precipitation^28^.

**Figure 4:**
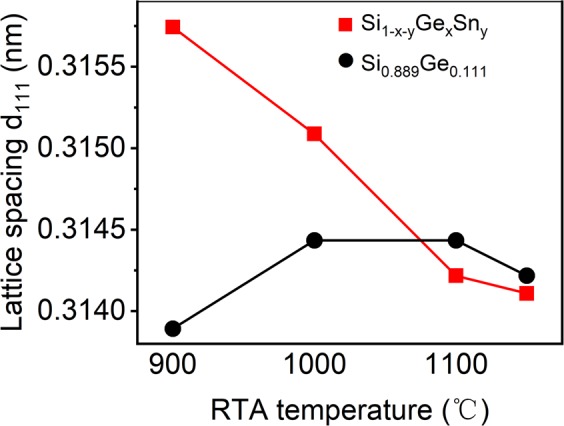
The RTA temperature dependence of the lattice distance for samples after 15 seconds-RTA.

Figure 4 shows the lattice distance *d*_111_ of ternary and binary alloy thin films as a function of the annealing temperature. The lattice distance was estimated from the diffraction peak position of the Bragg reflection. Comparison the tendency of Si_1-x-y_Ge_x_Sn_y_ and Si_0.889_Ge_0.111_, the turning point is 1000 °C. When the RTA temperature locates under 1000 °C, the evident smaller *d*_111_ lattice spacing of Si_0.889_Ge_0.111_ confirms the formation of ternary Si_1-x-y_Ge_x_Sn_y_ in Sn doping Si_1-x_Ge_x_ film, while when RTA temperature exceeds 1000 °C, the rapidly decline of lattice spacing *d*_111_ for Si_1-x-y_Ge_x_Sn_y_ samples suggests precipitation of Sn from ternary alloy.

**Figure 5:**
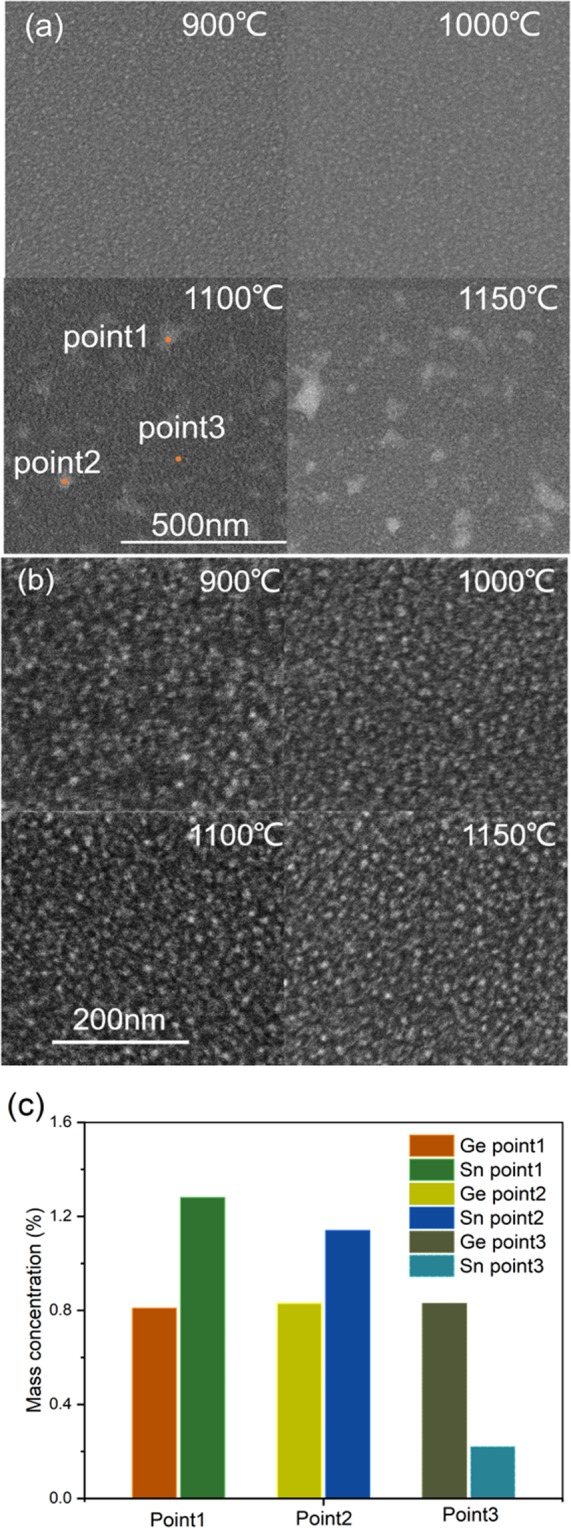
SEM images of (**a**) Si_1-x-y_Ge_x_Sn_y_ and (**b**) Si_0.889_Ge_0.111_ films after 15 s RTA. (**c**) Mass concentration of Ge and Sn atoms analyzed by EDS at point 1, 2, and point 3 in the Si_1-x-y_Ge_x_Sn_y_ sample after RTA at 1100 °C for 15 seconds.

Figure 5(a–c) show the surface SEM images and electron dispersive spectroscopy (EDS) analyses of Si_1-x-y_Ge_x_Sn_y_ and Si_0.889_Ge_0.111_ samples after RTA at various temperatures for 15 seconds, respectively. In Fig. 5(a), the uniform grain sizes and smooth surface are observed for 900 and 1000 °C -RTA samples. While non uniform metal Sn with average particles sizes of around 40 nm are clearly seen for 1100 °C post-annealing sample in Si_1-x-y_Ge_x_Sn_y_. The size of Sn particles increases to 60 nm for 1150 °C post-annealing sample. In Fig. 5(b) for a comparison, in Si_0.889_Ge_0.111_ samples, there are no any precipitation observed on the surface except the grain size increased from about 7 to 10 nm. This result agrees well with those of Raman and XRD. In Fig. 5(c), in order to eliminate the influence of Si and O_2_ content from the Si/SiO_2_ substrate, the changes of Ge and Sn ratios can be analyzed by EDS at each point, the ratios for Ge and Sn at points 1 and 2 of bright regions are estimated to be 0.83%, and 1.2%, respectively, while the ratio of Sn at point 3 of a darker region is just approximate 0.2%, which further verify that the Sn precipitates out when the annealing temperature exceeds 1100 °C, and the size of Sn particles increases rapidly with the annealing temperature.

**Figure 6:**
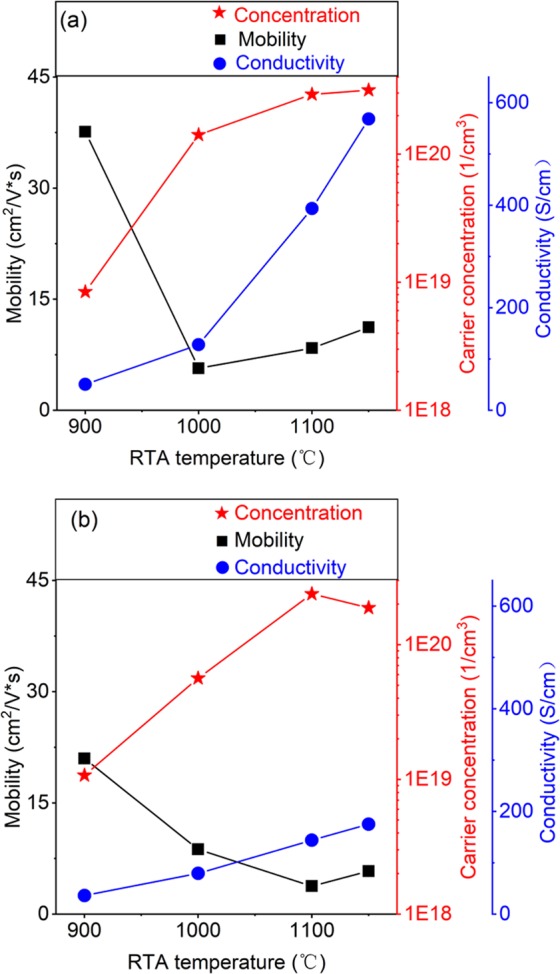
Hall measurement results (mobility, carrier concentration, and conductivity) of (**a**) Si_1-x-y_Ge_x_Sn_y_ and (**b**) Si_0.889_Ge_0.111_ samples after 15 seconds-RTA as a function of the RTA temperature.

Figure 6(a,b) show the Hall mobility, carrier concentration, and conductivity of samples annealed at different temperatures for Si_1-x-y_Ge_x_Sn_y_ and Si_0.889_Ge_0.111,_ respectively. The conductivity of Si_1-x-y_Ge_x_Sn_y_ samples increases faster than that of Si_0.889_Ge_0.111_ samples and when the annealing temperature over 1100 °C the mobility of Si_1-x-y_Ge_x_Sn_y_ samples are twice as large as corresponding Si_0.889_Ge_0.111_ samples. Moreover, the improved annealing temperature under conditions of carrier concentration of 10^20^ cm^−3^ order of magnitude which is contrary to the rule that the mobility decreases with the increase of carrier concentration^29^ as shown in Fig. 6(a,b), which attributing to suitable grain sizes and boundaries increase influence with heavy B doping^30,31^. After checking the carrier concentration data, one can find at least 900 °C-annealing is necessary for activation the implanted B ions^29,32^ for heavily doped samples of more than 10^15^ cm^−2^ dosage, furthermore annealing of more than 1000 °C, even if only for 15 seconds, can activate most of implanted B atoms, and even at the temperature of 1150 °C, almost all the implanted B atoms are activated which leading the measured Hall carrier concentration value is larger than the designed value 1.8 × 10^20^ cm^−3^.

**Figure 7:**
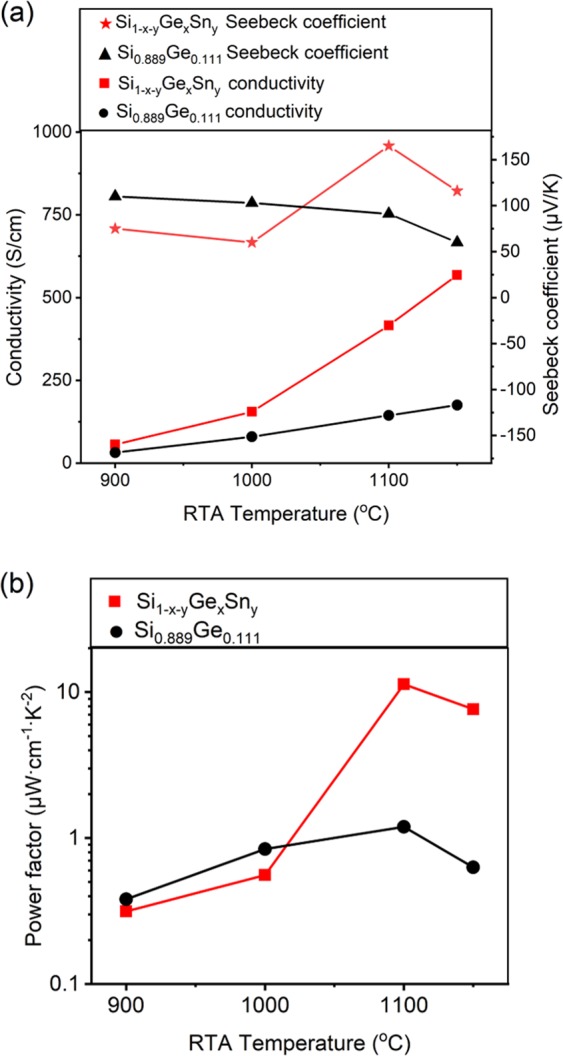
(**a**) The RTA temperature dependence of the Seebeck coefficient and conductivity of samples after 15 seconds-RTA. (**b**) The RTA temperature dependence of the power factor for samples after 15 seconds-RTA.

Figure 7(a,b) show the thermoelectric performance of Si_1-x-y_Ge_x_Sn_y_ and Si_0.889_Ge_0.111_ samples with same B dose. In Fig. 7(a), the conductivity of Si_1-x-y_Ge_x_Sn_y_ and Si_0.889_Ge_0.111_ samples improves with the increase of RTA temperatures, obviously the conductivity of Si_1-x-y_Ge_x_Sn_y_ increases faster than that of Si_0.889_Ge_0.111_ samples. One can find that after 1150 °C-RTA treated for 15 seconds, the maximum conductivity 175 S/cm of Si_0.889_Ge_0.111_ sample is less than a third of corresponding 568 S/cm of Si_1-x-y_Ge_x_Sn_y_ sample which is attributed to the high activation rate of implanted B atoms, the better crystallinity and larger grain size caused by Sn introduction. The increased carrier mobility revealed by Hall measurements is in good agreement with electrical conductivity results. All of the samples measured at room temperature showed positive Seebeck coefficients, mean the p-type semiconductors, which match the B implantation procedure well. Generally, with the higher carrier concentration over 10^20^ cm^−3^, improving carrier mobility is the most effective way to increase both electrical conductivity and Seebeck coefficient^33–35^. Therefore, the Seebeck coefficient and conductivity of Si_1-x-y_Ge_x_Sn_y_ samples are higher than the corresponding Si_0.889_Ge_0.111_ samples. And the maximum Seebeck coefficient of Si_1-x-y_Ge_x_Sn_y_ samples is 165 μV/K which is 1.8 times higher than a Si_0.75_Ge_0.25_ microcrystalline film doped with boron at 1.6 × 10^20^ cm^−3^, confirming the inverse dependence of Seebeck coefficient on carrier concentration^36^. Naturally, the maximum power factor *S*^*2*^*σ* value is calculated as 11.3 μWcm^−1^ K^−2^,which is about 1.5 times higher than the bulk Si_80_Ge_20_^37,38^. In Fig. 7(b), the highest power factor of the Si_0.889_Ge_0.111_ sample is one order of magnitude lower than the corresponding Si_1-x-y_Ge_x_Sn_y_ sample, which further proves that the introduction of Sn can greatly improve the thermoelectric property of Si_1-x_Ge_x_-based material. In stark contrast to our results, Paul *et al*.^39^ prepared nanoporous p-type Ca_3_Co_4_O_9_ thin films with a power factor of 2.3 μWcm^−1^ K^−2^ at room temperature, Zhou *et al*.^40^ reported a p-type copper telluride nanowires with a power factor of 0.23 μWcm^−1^ K^−2^ at RT, Park *et al*.^41^ prepared a p-type RTCVD graphene/PEDOT:PSS (RCG/P) hybrid film which power factor is 0.58 μWcm^−1^ K^−2^ at ∼300 K.

For some references, the thermal conductivity is likely to be even lower than that of Si_1-x_Ge_x_ in ternary Si_1-x-y_Ge_x_Sn_y_ alloys with increased mass and bond disorder^17,42^, the thermal conductivity is 1.1 W/mK for the sample annealed at 1100 °C for 15 second, matches with the literature^17^ value very well. The maximum *zT* value in this work can reach up to 0.31 at room temperature due to the super low thermal conductivity and the ultrahigh electrical conductivity.

## Conclusions

A champion material with key features such as abundance, low toxicity, biocompatibility polycrystalline Si_1-x-y_Ge_x_Sn_y_ films have been successfully deposited on Si/SiO_2_ wafers. The synthesized Si_1-x-y_Ge_x_Sn_y_ films have a microcrystalline grain structures ranging in size from 7 nanometers to 24 nanometers. It shows that the introduction of Sn into Si_1-x_Ge_x_ can significantly improve the conductivity of Si_1-x_Ge_x_ based thermoelectric materials at room temperature while obtaining a relatively high Seebeck coefficient. A high power factor value of 11.3 μW cm^−1^ K^−2^ has been achieved for optimized samples at room temperature, which is 5–50 times larger than TE performances of those reported p-type materials of Ca_3_Co_4_O_9_^39^, copper telluride nanowires^40^, RTCVD graphene/PEDOT:PSS (RCG/P) hybrid film^41^ at room temperature. The cost-effective and scalable techniques employed in this research point to future impact of the development of ambient thermoelectric materials both in academia and commercial.

## Methods

### Si_1-x-y_Ge_x_Sn_y_ film deposition

**Figure 8:**
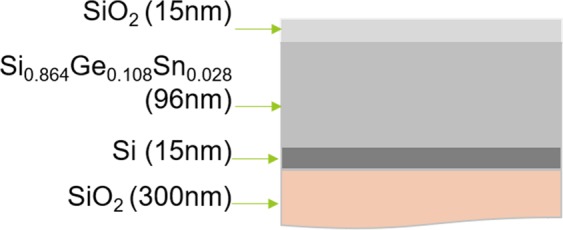
The schematic illustration of the cross-section sample structure.

The Si_0.864_Ge_0.108_Sn_0.028_ films were deposited by using a magnetron sputtering apparatus (MB6288, ULVAC Company) on Si/SiO_2_ wafers with a 300 nm-thick SiO_2_ layer. The base pressure was on the order of 1 × 10^−5^ Pa and the working pressure of Ar gas in the chamber was on the order of 0.1 Pa. Before the deposition, Si/SiO_2_ wafers were ultrasonically cleaned with absolute acetone, ethanol, and deionized water for 5 min in sequence, blow-dried by nitrogen, and then placed into the sample holder. The Si, Ge, and Sn compositions were accurately controlled by optimizing output power parameters of each sputter target. The purities of Si, Ge, and Sn targets were higher than 99.99%. After the deposition of Si_1-x-y_Ge_x_Sn_y_ thin film, a 15 nm-thick SiO_2_ layer was then sputtered on the top as a protective layer to avoid the oxidation. The structure of the film is shown in Fig. 8 and all of the sputtering parameters are summarized at the Table. 1 in the Supplementary Section.

### Ion implantation and RTA process

For improving the thermoelectric properties, the Si_0.864_Ge_0.108_Sn_0.028_ layers were implanted with B ion at a dose of 2 × 10^15^ atoms cm^−2^. The implantation energy of 16 keV was used to dope into the Si_0.864_Ge_0.108_Sn_0.028_ layer. The specific energies and the doses were selected for the ion implantation according to the calculation by using SRIM simulation. The details of the simulation are described in Supplementary Section. Subsequently a 15 seconds short-time RTA in N_2_ ambient was performed to electrically activate the dopants in the ternary alloy films, crystallize the Si_1-x-y_Ge_x_Sn_y_ thin film with annihilating defects and achieve a homogeneous doping profile through the whole thickness (AG association Heatpulse 610).

### Characterization and thermoelectric performance measurement

The crystal structure and crystallinity of Si_1-x-y_Ge_x_Sn_y_ layer were characterized using the X-ray diffraction (XRD) measurement (Rigaku RINT-2100) with Cu K_α1_ radiation with a wavelength of 1.54056 Å. The diffraction profiles were measured at a diffraction angle (2*θ*) from 20° to 65° with in a step of 0.02°. Micro Raman spectroscopy measurement was carried out using an excitation wavelength of 532 nm and a total laser power of 1.4 mW at room temperature (Nano photon Raman-11). Scanning electron microscopy (SEM Hitachi S-5200) was carried out at an acceleration voltage of 30 kV and a working current of 10 μA. The Hall mobility *μ*_н_ and carrier concentration *n* were measured using a Hall effect measurement system (Toyo corporation RESITEST 8300) at room temperature. The samples used for the measurement were cut into rectangular bars with approximate dimensions about of 1 × 1 cm^2^. Conductivity and Seebeck coefficient of samples were measured by using the SBA485 system (Netzsch), and the system errors were below about 7% and the measure temperature was at room temperature. The thermal conductivity of samples were measured using the ultrafast laserbased time-domain thermoreflectance (TDTR) method (Picotherm).

## Supplementary information

Supporting Information
