# Supplementary material for: Realizing High Thermoelectric Performance at Ambient Temperature by Ternary Alloying in Polycrystalline Si1-x-yGexSny Thin Films with Boron Ion Implantation
Source: Sci Rep. 2019 Oct 4;9:14342. doi: 10.1038/s41598-019-50754-4 (PMC6778188; doi:10.1038/s41598-019-50754-4)
Supplement: Supplementary file 1 — Supporting Information [file 41598_2019_50754_MOESM1_ESM.pdf]

Supplementary

Title: Realizing High Thermoelectric Performance at Ambient Temperature by Ternary Alloying in Polycrystalline $Si_{1-x-y}Ge_xSn_y$ Thin Films with Boron Ion Implantation

Author: Ying Peng, Lei Miao, Jie Gao, Chengyan Liu, Masashi Kurosawa, Osamu Nakatsuka, and Shigeaki Zaima

1. Thermal conductivity measurement

The ultrafast laserbased time-domain thermoreflectance (TDTR) technology, front heating front detection (FF) mode schematic diagram.

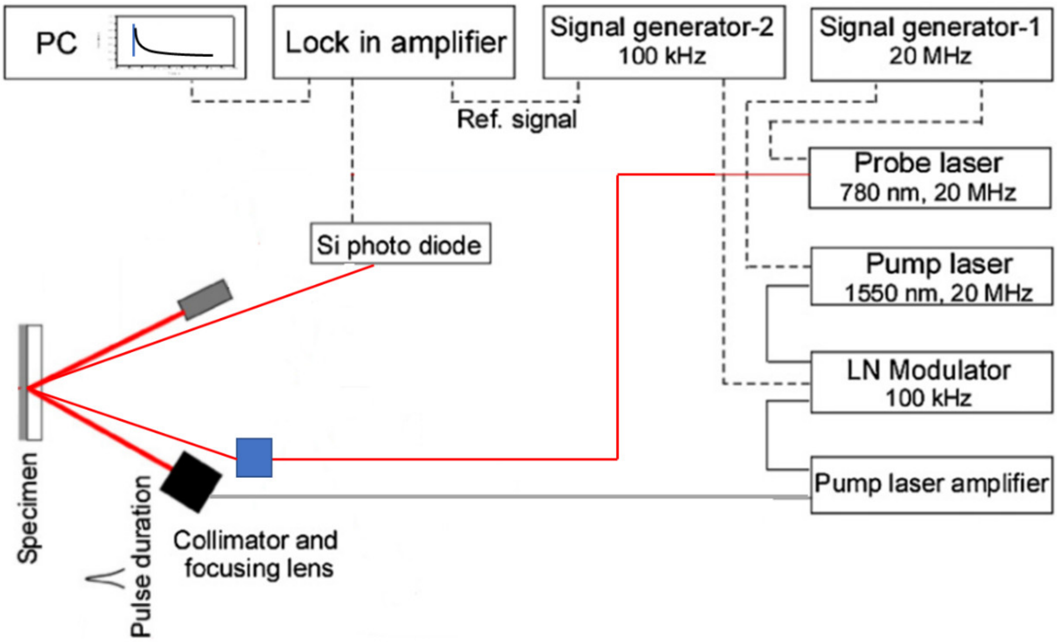

Figure 1. Schematic diagram of the FF laser pulsed thermoreflectance system.

Analysis test curve shown in Figure 2, use the formula[1]

$$T_f(t) = \frac{1}{b_f\sqrt{\pi t}}\left[1 + 2\sum_{n=1}^{\infty} \gamma^n \exp\left(-n^2 \frac{\tau_f}{t}\right)\right]$$

Get the sample thermal permeability $b= 1332\ Js^{-0.5}m^{-2}k^{-1}$

Use $\kappa= b^2/\rho c$, here for $Si_{0.864}Ge_{0.108}Sn_{0.028}$, $\rho=2786 kg/m^3$, $c=587.8 J/kgK$

Get thermal conductivity $\kappa=1.1 W/mk$

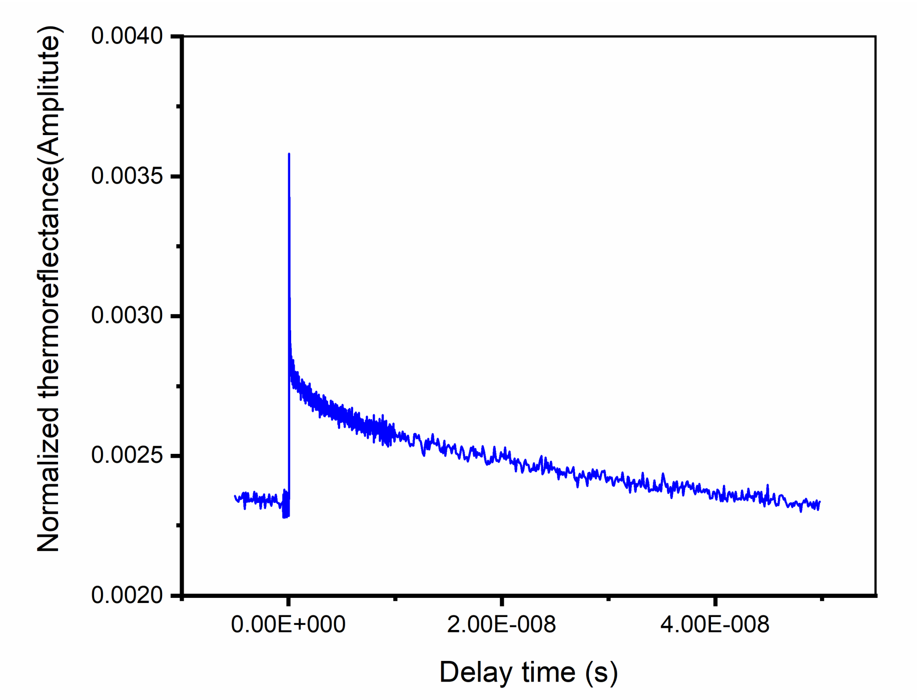

Figure 2. Temperature history curve of the 100 nm Mo film on 1100 deg C RTA $Si_{0.864}Ge_{0.108}Sn_{0.028}$ film.

2. SRIM ion implantation simulation

The SRIM simulation was performed for layers of the structure shown in Figure 3. First the projected range versus implantation energy was computed for the Boron ions in the amorphous $Si_{0.864}Ge_{0.108}Sn_{0.028}$ material to determine the nominal implantation energies (16 KeV) to be used. Before implantation, the simulated carrier concentration of the amorphous $Si_{0.864}Ge_{0.108}Sn_{0.028}$ is about 9×10^{4} atoms cm^{-2}, then with appropriate dose numbers 2×10^{15} atoms cm^{-2}, the simulative maximal concentration is approximately 1.8×10^{20} atoms cm^{-2}.After implantation, 15-second 900, 1000, 1100 and 1150 °C rapid annealing process was chosen to regulate the distribution of carrier concentration from 10^{19} to10^{20} /cm^{3}.

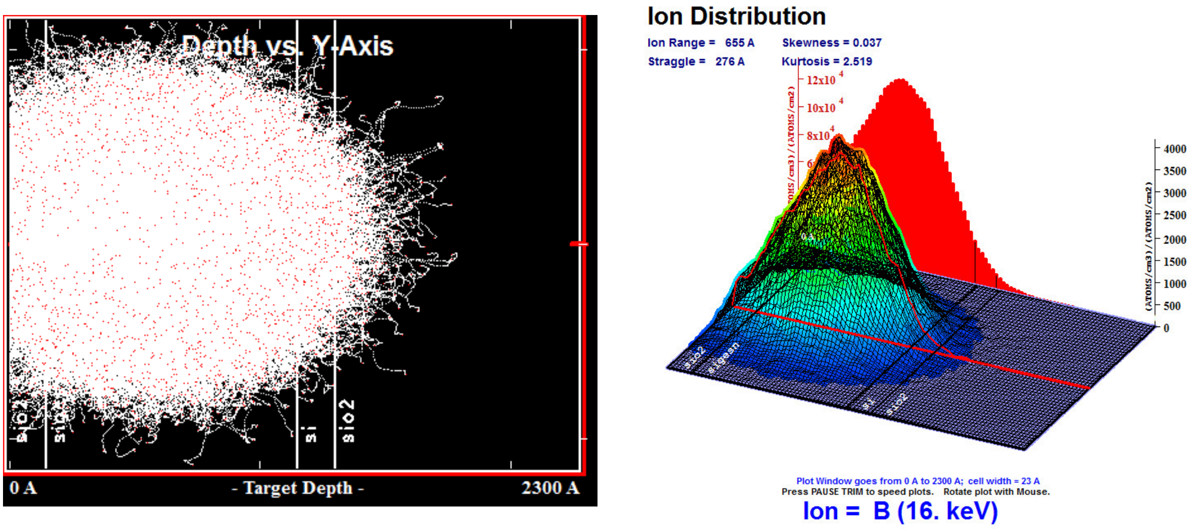

Figure 3. Simulated B-ion implantation depth and distribution profile in $Si_{1-x-y}Ge_{x}Sn_{y}$ layer.

Table1. The sputtering parameters for depositing Si, $Si_{0.864}Ge_{0.108}Sn_{0.028}$, and SiO_2 films

**Deposited layer**	**RF power (W)**	**Deposition rate (nm/min)**	**Deposition time(s)**	**Thickness (nm)**
**Si buffer**	Si 150	2.80	313	15
**$Si_{0.864}Ge_{0.108}Sn_{0.028}$**	Si 150<br>Ge 10<br>Sn 3	2.80<br>0.35<br>0.09	1790	96
**SiO_2**	SiO_2 150	2.92	309	15

Reference

1. Baba, T. Analysis of One-dimensional Heat Diffusion after Light Pulse Heating by the Response Function Method. *Japanese Journal of Applied Physics* **48**, 05EB04 (2009).
